# Development and Application of KASP Markers for Candidate Glucosinolate Biosynthesis Genes in Broccoli

**DOI:** 10.3390/ijms27062714

**Published:** 2026-03-16

**Authors:** Sifan Du, Yusen Shen, Mengfei Song, Xiaoguang Sheng, Huifang Yu, Shuting Qiao, Jiaojiao Li, Honghui Gu, Zihong Ye, Jiansheng Wang

**Affiliations:** 1 College of Life Sciences, China Jiliang University, Hangzhou 310018, China; 2 Institute of Vegetables, Zhejiang Academy of Agricultural Sciences, Hangzhou 310021, Chinaguhh@zaas.ac.cn (H.G.); 3 Department of Vegetable Science, College of Horticulture, China Agricultural University, Beijing 100193, China; 4 Department of Horticultural Science, Zhejiang Agriculture and Forestry University, Hangzhou 311300, China

**Keywords:** KASP marker, *AOP2*, *GSL-OH*, GNA, PRO, GRA, broccoli

## Abstract

Broccoli is rich in glucosinolates (GSLs), secondary metabolites that contribute to both plant defense and human health. Optimizing the composition of major aliphatic GSLs is an important breeding objective, yet robust molecular markers for marker-assisted selection (MAS) remain limited. In this study, candidate gene-based kompetitive allele-specific PCR (KASP) markers were developed from conserved GSL biosynthesis genes, focusing on *AOP2* and *GSL-OH* selected from 19 GSL-related genes. Marker–trait associations were evaluated in a natural broccoli population and further validated in an independent F_2_ population. Among the tested markers, S101, located in *AOP2*, exhibited consistent genotype-dependent effects on GNA and PRO across both populations, supporting its stable predictive value. Receiver operating characteristic (ROC) analysis further confirmed strong classification performance of S101 for distinguishing high- and low-content genotypes of these traits in the F_2_ population. In contrast, S074 and *S035* showed population-dependent effects, with significant associations detected only in the natural population. Although association signals were reduced under mixed linear model (MLM) analysis with false discovery rate (FDR) correction, major loci identified under the general linear model (GLM) framework remained detectable. Overall, these results demonstrate the potential of candidate gene-based KASP markers for improving aliphatic GSL composition in broccoli through marker-assisted selection.

## 1. Introduction

Broccoli (*Brassica oleracea* L. var. *italica*) is rich in glucosinolates (GSLs), a class of secondary metabolites with significant anticancer and antioxidative activities that also play crucial roles in plant defense against biotic and abiotic stresses [1,2]. Different GSL components exhibit distinct biological functions and nutritional values. Considerable natural variation in both total GSL content and individual GSL composition has been reported among broccoli genotypes. Recent metabolomic studies [3] have further demonstrated complex accumulation patterns of nutrients and GSLs across different genotypes and related *Brassica* vegetables, highlighting substantial genetic diversity and emphasizing the importance of elucidating the genetic basis underlying GSL variation. Among individual GSLs, glucoraphanin (GRA) is a desirable compound with well-documented health-promoting properties, whereas progoitrin (PRO) is considered undesirable due to its potential adverse effects [4,5]. Gluconapin (GNA) occupies a central metabolic position in the aliphatic GSL pathway, serving as a key intermediate linking GRA biosynthesis to PRO formation [6]. Consequently, modulating the balance between GRA, GNA, and PRO has become a major objective in broccoli-breeding programs aimed at improving nutritional quality.

GSLs are classified into aliphatic, aromatic, and indole types based on their precursor amino acids, with aliphatic and indole GSLs being predominant in broccoli. GSL biosynthesis involves amino acid side-chain elongation, formation of the core structure, and subsequent side-chain modifications, with multiple structural and regulatory genes coordinately controlling aliphatic side-chain composition [7,8,9]. Increasing evidence from genetic and association studies has demonstrated that variation in key biosynthetic genes contributes significantly to natural differences in GSL composition among broccoli genotypes [10]. Within the aliphatic GSL pathway [11], *AOP2* and *GSL-OH* play pivotal roles in determining four-carbon side-chain composition by catalyzing the conversion of GRA to GNA and GNA to PRO, respectively. These enzymatic steps provide a clear molecular basis for targeting specific GSL components through candidate gene-based marker development. In addition to aliphatic GSLs, indole GSLs such as 4-hydroxyglucobrassicin (4HGBS) contribute to overall GSL variation and are involved in plant defense and stress responses, further highlighting the importance of profiling both aliphatic and indole GSLs in breeding studies aimed at improving nutritional and functional quality [12].

Currently, high-performance liquid chromatography (HPLC) is widely used for determining GSL levels due to its high accuracy and cost-effectiveness [13]; however, direct measurement of GSL content is time-consuming and labor-intensive and influenced by environmental conditions, making it unsuitable for large-scale screening in breeding populations. This limitation underscores the need for molecular markers that can reliably predict GSL variation across genotypes. Early genetic studies using genome-anchored single-nucleotide polymorphism (SNP) markers have demonstrated that aliphatic GSL composition in broccoli is a quantitatively inherited trait controlled by multiple loci, with significant SNP–trait associations detected for major compounds [14]. These findings highlight the potential of SNP-based approaches for dissecting the genetic architecture of GSL variability and provide a foundation for marker-assisted selection in breeding programs.

Among SNP-based genotyping methods, kompetitive allele-specific PCR (KASP) have gained popularity due to their high efficiency, accuracy, and stability, making them ideal for genotyping large populations with a limited number of target loci [15,16]. KASP assays have been successfully developed for crops such as cauliflower, cabbage, and soybean [17,18,19]. In broccoli, however, KASP marker development has been mainly focused on varietal identification and genetic characterization [16], with relatively few markers functionally validated for GSL content variation and marker-assisted selection (MAS).

To address this limitation, we employed a candidate gene-based association mapping approach using re-sequencing data from 23 broccoli accessions to identify SNPs within 19 key GSL biosynthesis genes. Based on functional annotation and genomic positions, representative and potentially functional SNPs were selected for KASP marker development. These KASP markers were subsequently evaluated in a panel of 106 broccoli accessions to assess their associations with major aliphatic GSLs (GNA, PRO, GRA) as well as the indole GSL 4HGBS. By integrating biologically informed candidate gene selection with population-based validation, this study aims to develop and evaluate functionally relevant molecular markers associated with GSL variation, thereby providing a foundation for future marker-assisted breeding efforts in broccoli.

## 2. Results

### 2.1. Development of KASP Markers for GSLs Genes in Broccoli

In this study, 89 *Arabidopsis* genes related to GSL metabolism were obtained from the *Brassica* Database (BARD http://Brassicadb.cn (accessed on 10 January 2026)) and used as queries for NCBI BLAST (https://blast.ncbi.nlm.nih.gov, accessed on 10 January 2026) against the HDEM broccoli reference genome [6,20]. After applying similarity-based filtering and redundancy removal, 293 GSL-related homologs (Table S1) were identified in broccoli, consistent with gene expansion following polyploidization from *Arabidopsis thaliana* to *Brassica oleracea*.

Previously, a total of 23 diverse broccoli genotypes were used for whole-genome re-sequencing, and a million numbers of SNPs were detected [21]. From this SNP platform, 1276 non-synonymous SNPs located in the exon region of the broccoli’s GSLs-related genes were screened, which may affect the GSLs composition or contents (Table S1).

**Figure 1:**
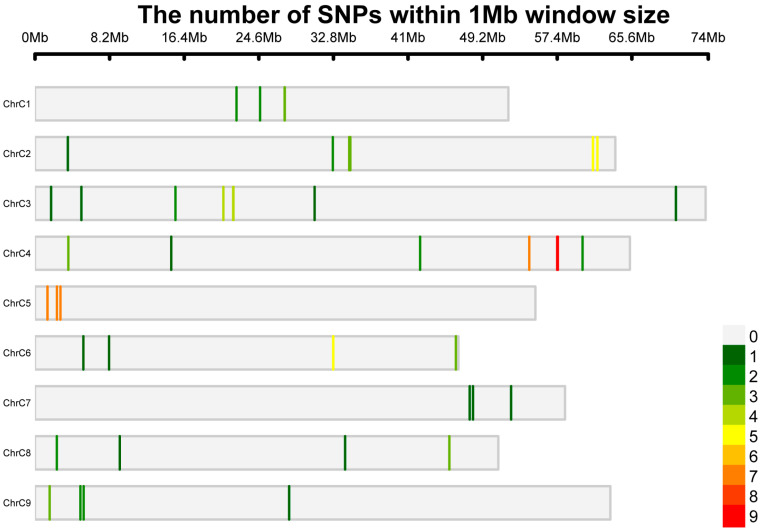
The density distribution of 97 SNPs on the chromosome. Chromosomal distribution and SNP density of the 97 SNPs across the broccoli HDEM genome, calculated using a 1 Mb sliding window. The horizontal axis indicates the physical position (in megabases, Mb) along each chromosome, spanning from 0 Mb to 74 Mb. The color gradient (from white to red) represents the number of SNPs per 1 Mb window, with values ranging from 0 (white) to 9 (red) as shown in the color scale.

For KASP markers, we performed multi-dimensional screening of candidate SNPs. For the identified SNP loci, those with no other polymorphic sites within 50 base pairs upstream and downstream, as well as those whose GC content of the primers is greater than 30% were selected [22]. A total of 108 candidate SNPs were selected for KASP primer design targeting key genes involved in GSL biosynthesis in broccoli, including *AOP2*, *AOP3*, *GSL-OH*, *MYB28*, *MYB29*, and *BCAT3*. Genotyping consistency was first evaluated across 23 core germplasms, resulting in the establishment of a KASP marker platform comprising 108 putative markers. After quality control, 97 markers showing high genotyping quality and reproducibility were retained (89.81%) and subsequently used for association analysis with GSL-related traits (Table S2). To assess the distribution of 97 SNPs along the chromosome, we plotted the SNP density distribution within a 1 Mb window of the HDEM genome of broccoli (Figure 1). It is noteworthy that the region on chromosome C9 and C3 exhibited a relatively low SNP density across the genome, and chromosome C5 exhibited a higher frequency. The established marker platform provides a reliable foundation for subsequent association analysis of GSLs-related candidate genes in broccoli.

### 2.2. Determination of GSL Contents in Broccoli

**Figure 2:**
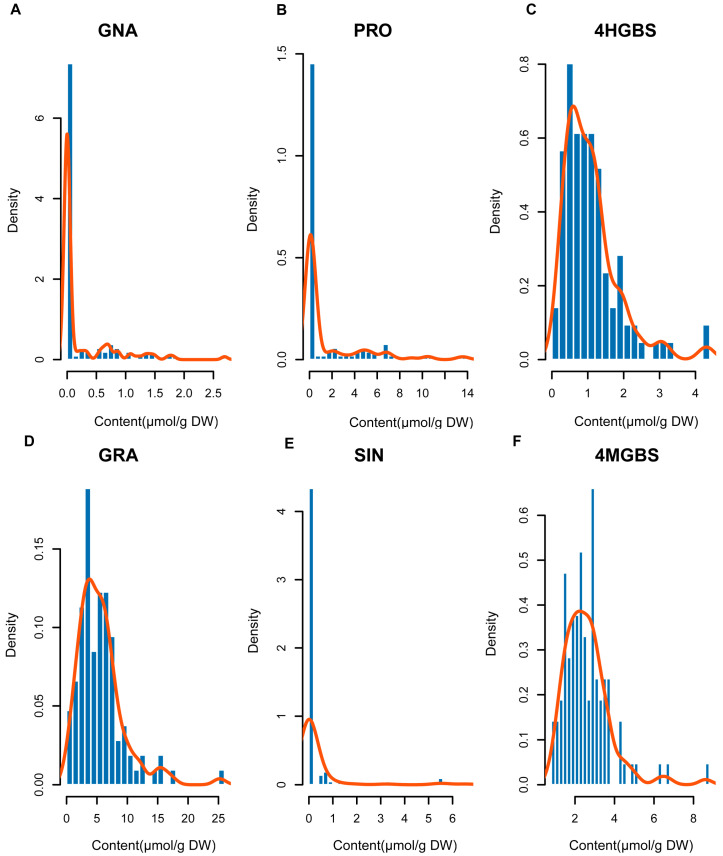
Phenotypic distribution and correlation analysis of GSLs components in broccoli. (**A**–**F**) Content distribution histograms (bars) and kernel density curves (lines) for six aliphatic GSL components: (**A**) GNA, (**B**) PRO, (**C**) 4HGBS, (**D**) GNA, (**E**) PRO, and (**F**) 4HGBS. The x-axis represents GSL content (μ mol/g dry weight, DW), and the y-axis indicates kernel density values. Density curves were fitted using a Gaussian kernel function based on measurements from 106 broccoli accessions.

The content and composition of GSLs in broccoli florets were detected by using the HPLC method. The GSL composition in broccoli florets was similar to that reported in previous studies [23], with seven aliphatic GSLs and three indole GSLs preliminarily identified (Tables S3 and S4). Six representative GSL components were further analyzed based on their biological importance (Table 1), including four aliphatic GSLs (GRA, GNA, PRO, and SIN) and two indolic GSLs (4HGBS and 4MGBS). These traits were chosen based on their key positions in the GSL biosynthetic pathway, their phenotypic variability in the population, and their relevance to nutritional and breeding value. The phenotypic distributions of all six traits are shown in Figure 2.

Among the aliphatic GSLs, GNA and PRO showed continuous and approximately normal distributions across the population, a characteristic typical of quantitatively varying metabolic traits. GNA (Figure 2A) showed substantial phenotypic variation (CV = 2.08), whereas PRO (Figure 2B) showed slightly lower dispersion (CV = 1.89). The indolic GSL 4HGBS (Figure 2C) displayed a relatively low coefficient of variation (CV = 0.72) with a more concentrated distribution. Similarly, GRA (Figure 2D) exhibited moderate variability (CV = 0.69) across the population. In contrast, SIN (Figure 2E) showed a right-skewed distribution toward lower values and the highest variability among the examined traits (CV = 3.78), indicating pronounced heterogeneity across accessions. 4MGBS (Figure 2F) displayed the lowest coefficient of variation (CV = 0.46) and a relatively concentrated distribution pattern, reflecting stable accumulation within the population.

**Figure 3:**
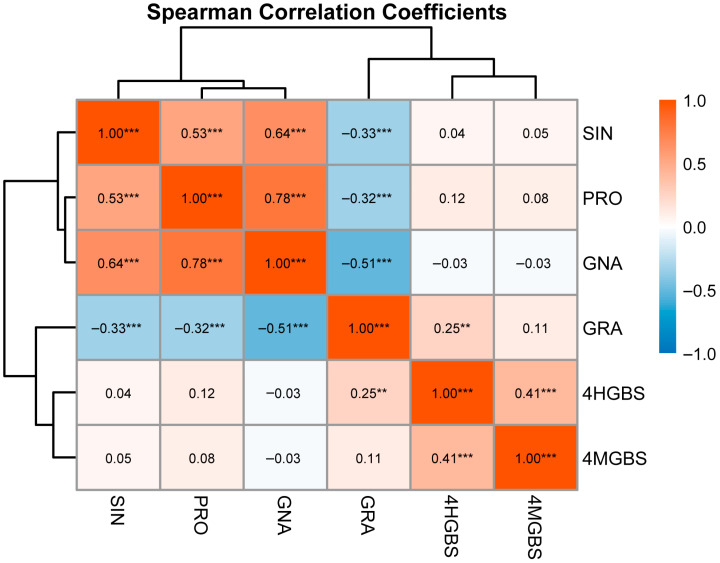
Spearman correlation coefficient heatmap for different GSL contents. This figure shows a heatmap of Spearman correlation coefficients among GSL traits (SIN, PRO, GNA, GRA, 4HGBS, and 4MGBS). Data are presented for the natural population (*n* = 106). Color intensity reflects the strength and direction of correlations, ranging from negative (blue) to positive (red). Hierarchical clustering dendrograms were generated based on the Spearman correlation matrix, with shorter branch lengths indicating stronger monotonic associations and grouping of traits with similar correlation patterns. **, *p* ≤ 0.01; ***, *p* ≤ 0.001.

Correlation analysis revealed distinct relationships among the examined GSL traits (Figure 3). A strong positive correlation was observed between GNA and PRO (*r* = 0.78, *p* < 0.001). SIN was positively correlated with both GNA (*r* = 0.64, *p* < 0.001) and PRO (*r* = 0.53, *p* < 0.001). In contrast, GRA showed significant negative correlations with GNA (*r* = −0.51, *p* < 0.001) and PRO (*r* = −0.32, *p* < 0.001). Among indolic GSLs, 4HGBS and 4MGBS were moderately positively correlated (*r* = 0.41, *p* < 0.001). Correlations between aliphatic and indolic GSLs were weak and generally non-significant (|*r*| < 0.12).

Overall, aliphatic GSLs exhibited coordinated variation patterns with evidence of both positive and negative relationships within the GRA-GNA-PRO pathway, whereas correlations between aliphatic and indolic GSLs were generally weak. These findings indicate metabolic differentiation between GSL classes in the natural population.

### 2.3. Identification and Validation of Candidate SNP Markers Associated with GSL Traits

#### 2.3.1. Identification of Candidate SNP Markers in the Natural Population

**Figure 4:**
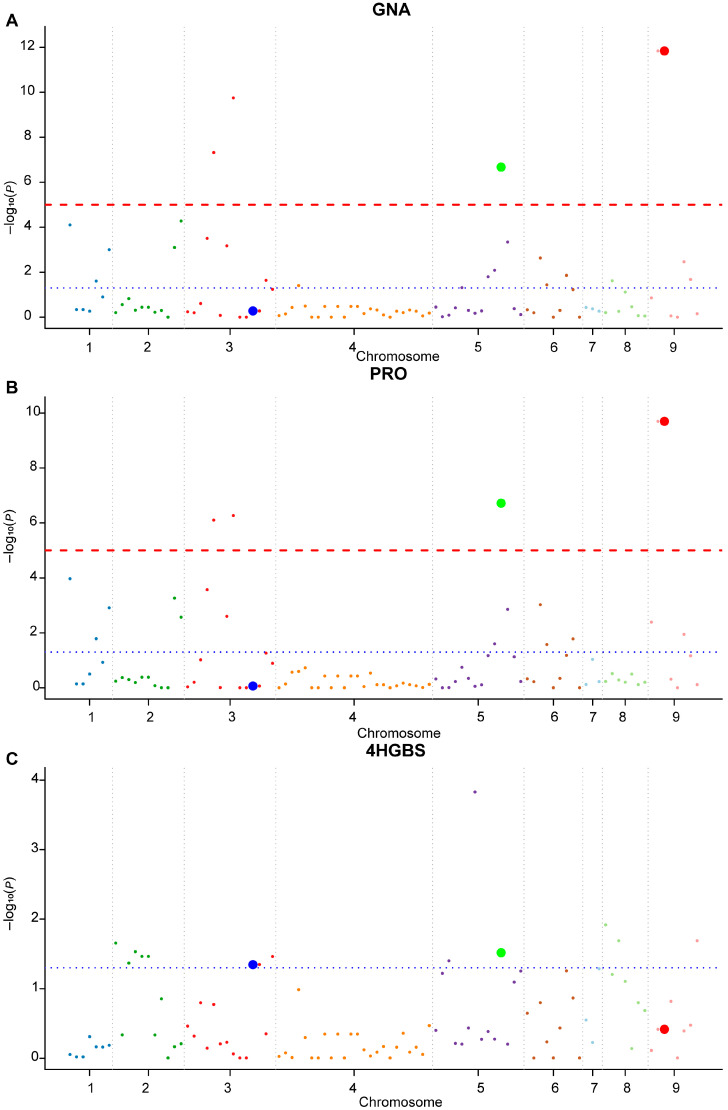
Manhattan plots of candidate gene-based association analysis for GNA, PRO, and 4HGBS contents in 106 broccoli accessions in GLM. (**A**) GNA; (**B**) PRO; (**C**) 4HGBS. The x-axis represents the physical positions of SNPs along the nine broccoli chromosomes (bp), and the y-axis represents the −log_10_(*p*) values. Each point represents an SNP, and there are a total of 97 SNPs. Alternating colors are used to distinguish the nine broccoli chromosomes for better visualization. Red bold dots represent S101, green bold dots represent S074, and blue bold dots represent S035. Two significance thresholds are shown: a blue dashed line indicates *p* = 5 × 10^−2^, and a red dashed line indicates *p* = 1 × 10^−5^. Detailed association results including chromosomal position, *p* values, and proportion of PVE are provided in Table S5.

To identify robust KASP markers associated with GSL content in broccoli, 97 high-quality SNPs derived from candidate genes involved in GSL biosynthesis were subjected to candidate gene association analysis in 106 broccoli accessions. Genotype–phenotype associations were analyzed using a general linear model (GLM) implemented in TASSEL (Version 5.0; Bioinformatics Research Center, Iowa State University, Ames, IA, USA) [24] (Tables S5 and S6). SNPs were considered candidate loci when they met a nominal significance threshold (*p* < 0.05) and explained more than 5% of the phenotypic variance (*R*^2^ > 5%). Based on these criteria, nine SNPs were significantly associated with six GSL-related traits (Figure 4 and Figure S1). The nine significant SNPs were distributed across chromosomes C3, C4, C5, and C9, with phenotypic variance explained (PVE) ranging from 5.19% to 38.37%. The strongest signals were detected on chromosome C9, where S100 and S101 showed the highest PVE for GNA (38.37%) and PRO (32.35%) (Table S6). Several associated SNPs were located within coding regions of known aliphatic GSL biosynthesis genes, including *AOP2*, *AOP3*, and *GSL-OH*, supporting the biological relevance of the detected associations.

**Figure 5:**
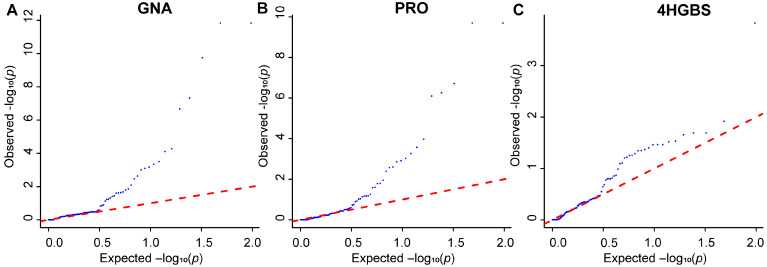
QQ plots of 97 SNPs associated with GNA, PRO, and 4HGBS in GLM. (**A**) GNA; (**B**) PRO; (**C**) 4HGBS. Each point represents an SNP, and there are a total of 97 SNPs. The x-axis represents the expected −log_10_(*p*) values, and the y-axis represents the observed −log_10_(*p*) values. The blue dots represent the observed association signals, and the red line represents the theoretical null distribution (y = x). Comparing observed and expected −log_10_(*p*) values to assess the overall distribution of association signals. Detailed association statistics are provided in Table S5.

Quantile–quantile (QQ) plots under the GLM revealed deviations from the expected null distribution for certain traits, particularly GNA, PRO, and SIN (Figure 5 and Figure S2), suggesting potential inflation due to population structure. To address multiple testing, false discovery rate (FDR) correction was applied using the Benjamini–Hochberg (BH) procedure, and adjusted *q*-values are provided in Table S5. Given the candidate gene design and moderate sample size, selected loci were subsequently subjected to validation in an independent F_2_ population.

To account for population structure and relatedness, a mixed linear model (MLM) incorporating PC1-PC3 and a kinship matrix was applied. Compared with GLM results, MLM analysis substantially reduced the number of significant associations, reflecting stricter control of false positives (Figures S3–S5). Nevertheless, several key loci identified by GLM remained detectable under MLM. In particular, S101 on chromosome C9 retained significant associations with GNA and PRO, although the explained variance decreased (5.13–9.03%) (Tables S7 and S8). QQ plots under the MLM showed improved conformity to the expected distribution (Figure S5), indicating effective control of population structure and relatedness. Given the candidate gene design and the moderate number of tested loci, marker prioritization was based on integrated evidence, including statistical significance across models, biological relevance within the candidate gene pathway, and subsequent validation in an independent F_2_ population.

**Figure 6:**
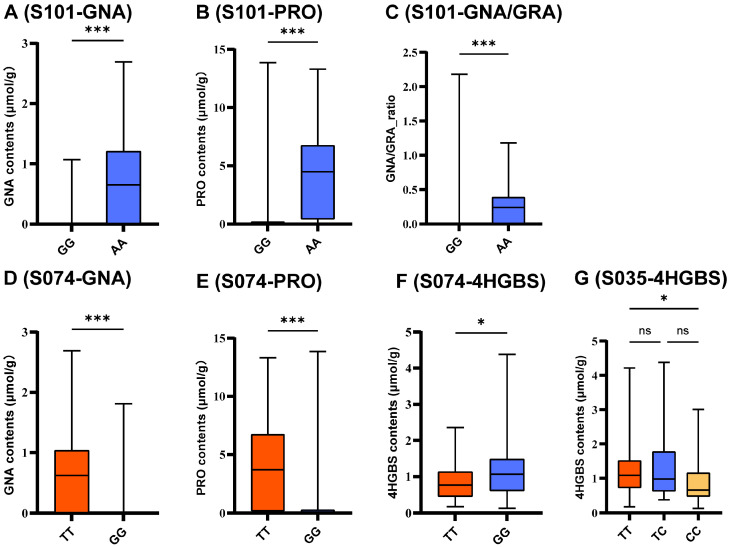
Genotype–phenotype associations of key KASP markers in a natural broccoli population. (**A**–**C**) Associations of S101 with GNA, PRO, and GNA/GRA content, respectively. (**D**–**F**) Associations of S074 with GNA, PRO, and 4HGBS, respectively. (**G**) Association of S035 with GNA. Box plots show the phenotypic variation in aliphatic GSLs among different genotypes. S101: Chr9: 1,616,635; S074: Chr5: 2,407,717; S035: Chr3: 21,810,653; Data are presented for the natural population (*n* = 106). Boxes represent the interquartile range (IQR), horizontal lines indicate median values, and whiskers denote the minimum and maximum values. Different letters indicate significant differences among genotypes. Significance levels are indicated as: ns, not significant; *, *p* ≤ 0.05; ***, *p* ≤ 0.001. Detailed statistics are provided in Table S11.

Genotype-stratified boxplot analysis was conducted for three representative SNPs (S101, S074, and S035) to evaluate phenotypic differentiation (Figure 6 and Figure S6). Clear genotype-dependent differences were observed for key breeding-related traits, including GNA, PRO, GRA, and 4HGBS. S101 exhibited consistent effects on GNA and PRO, S074 influenced multiple aliphatic GSL components, whereas S035 showed more trait-specific effects. For clarity, major traits are presented in the main text, with additional results provided in Figure S6 and Table S11.

**Figure 7:**
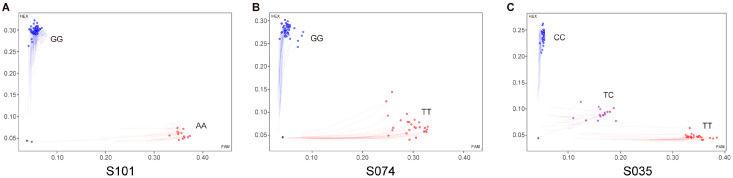
KASP genotyping results of markers S101, S074, and S035 in the natural population. Each data point represents an individual sample, with positions determined by fluorescence signals. Black dots represent the non-template control (NTC). (**A**–**C**) Scatter plots showing genotype clustering for markers S101, S074, and S035, respectively. (**A**) For S101, the alleles were A and G, forming two clusters corresponding to the genotypes AA (red) and GG (blue). (**B**) For S074, the alleles were T and G, forming clusters of genotypes TT (red) and GG (blue). (**C**) For S035, the alleles were C and T, forming clusters of genotypes TT (red), CC (blue), and TC (purple).

Linkage disequilibrium (LD) analysis was performed to evaluate redundancy among associated SNPs (Figure S7 and Table S10). Strong LD was observed among several loci located on the same chromosomes. On chromosome C9, S100 and S101 were in complete LD (*R*^2^ = 1.00, D′ = 1.00), and S101, located within *AOP2*, was retained as the representative marker. On chromosome C3, *S035* and S036 were highly linked in LD (*R*^2^ = 0.96, D′ = 0.98) and both associated with 4HGBS; S035 was selected over closely linked loci due to its higher PVE. Similarly, among SNPs within the *GSL-OH* region on chromosome C5, S072-S075 formed a moderately linked block (*R*^2^ = 0.30–0.76; D′ = 0.69–1.00); S074 exhibited the strongest and most consistent associations and was prioritized. Based on association strength, LD structure, and biological relevance, three representative KASP markers—S101, S074, and S035—were selected for subsequent validation. Genotyping results for these markers are shown in Figure 7, and corresponding allelic effect estimates are provided in Table S9.

#### 2.3.2. Validation of Candidate SNP Markers in the F_2_ Population

To validate the effectiveness of KASP markers identified in the natural population, an F_2_ population was developed from a cross between B019 (paternal line) and B109 (maternal line), which were selected based on contrasting GSL profiles (Table S3). Specifically, B019 exhibited high GNA content, whereas B109 showed low or undetectable levels of GNA and PRO, ensuring sufficient phenotypic divergence for segregation analysis. Eight independent GSL components were detected in the F_2_ population (Tables S13 and S14). Among them, GNA, PRO, GRA, and 4HGBS displayed continuous distributions with substantial phenotypic variation (Table 2), consistent with quantitative inheritance and suitable for marker validation. In contrast, 4MGBS and SIN showed inconsistent detection across individuals and were therefore excluded from subsequent validation analyses.

**Figure 8:**
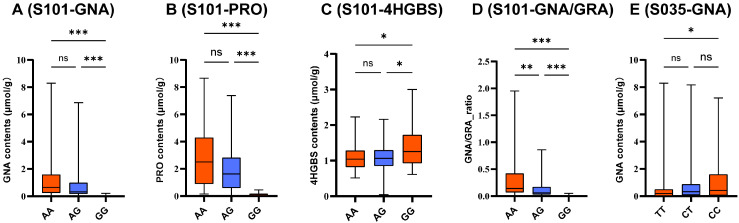
Genotype–phenotype associations of key KASP markers in a F_2_ broccoli population. (**A**–**D**) Associations of S101 with GNA, PRO, 4HGBS, and GNA/GRA content, respectively. (**E**) Associations of S035 with GNA. Box plots show the phenotypic variation in GSLs among different genotypes. S101: Chr9: 1,616,635; S035: Chr3: 21,810,653; Data are presented for the F_2_ population (*n* = 189). Boxes represent the interquartile range (IQR), horizontal lines indicate median values, and whiskers denote the minimum and maximum values. Different letters indicate significant differences among genotypes. Significance levels are indicated as: ns, not significant; *, *p* ≤ 0.05; **, *p* ≤ 0.01; ***, *p* ≤ 0.001. Detailed statistics are provided in Table S12.

Genotyping of the F_2_ population was conducted using three representative KASP markers (S101, S074, and S035), and association analyses were performed using reliably detected GSL traits. Genotype-based boxplot analysis illustrated clear phenotypic differentiation for selected marker–trait combinations (Figure 8 and Figure S8). S101 showed highly significant associations with both GNA and PRO in the F_2_ population, consistent with results from the natural population. Clear genotype-dependent differences were observed for GNA, and similar directional effects were detected for PRO, supporting the biological relevance of this locus. Receiver operating characteristic (ROC) analysis further demonstrated strong predictive performance of S101 (Table 3 and Figure S9). The marker correctly classified 93.88% of high-GNA and 90.70% of low-GNA individuals, as well as 89.80% of high-PRO and 97.67% of low-PRO individuals. In contrast, predictive performance for other traits was limited (Table 3), with detailed information shown in Table S12. These results indicate that S101 exhibits strong discriminatory power for major aliphatic GSL components within the F_2_ population. In contrast, S074 exhibited population-dependent effects. Although significantly associated with multiple GSL traits in the natural population, no consistent associations were detected in the F_2_ population, suggesting potential genetic background dependence or local LD structure. S035 displayed moderate predictive ability for GNA, correctly identifying 60.53% of high-content and 65.63% of low-content individuals, indicating limited practical utility. Collectively, validation in the F_2_ population confirmed the reproducible and strong predictive value of S101 for GNA and PRO, whereas S074 and S035 exhibited population- or trait-specific effects. These findings highlight the necessity of independent population validation prior to deployment in marker-assisted selection.

### 2.4. Variation Analysis of AOP2 and GSL-OH Gene in Broccoli

Previous studies have established *AOP2* and GSL-OH as key regulators of aliphatic GSL biosynthesis in *Brassica* crops [6]. Based on association signals identified in the natural population (Table S8), sequence variations within these candidate genes were further examined to assess their potential relevance to phenotypic variation.

KASP marker S101, located within an exon of *AOP2* on chromosome C9, represents an A/G polymorphism. In the natural population, KASP marker S101 displayed genotype-dependent effects on multiple aliphatic GSL components, with the strongest trends observed for GNA and PRO. These patterns suggest that S101 may influence the natural variation in the GRA-GNA-PRO pathway, contributing to coordinated changes in multiple metabolites rather than affecting a single compound. Although statistical significance was reduced under more conservative models, directional effects were consistently evident across genotypes, particularly for GNA and PRO. Evaluation in the F_2_ population further supported the functional relevance of S101. Clear genotype-dependent differences were observed for GNA and PRO, with effects largely consistent with the patterns detected in the natural population. The marker demonstrated strong predictive potential for distinguishing high- and low-content individuals for these traits, whereas its influence on other GSL components was comparatively moderate.

KASP marker S074, located within an exon of the *GSL-OH* gene on chromosome C5, represents a T/G polymorphism. In the natural population, S074 was associated with variation in PRO and GRA, suggesting a potential role in modulating aliphatic GSL composition. However, this association was not reproduced in the F_2_ population, indicating that the effects of S074 may be influenced by genetic background or population-specific factors.

Similarly, KASP marker S035, located within an exon of *AOP2* on chromosome C3, exhibited context-dependent trait associations. In the natural population, S035 was primarily associated with 4MGBS, whereas in the F_2_ population, its effects were observed on GNA content. This shift in trait associations suggests that the phenotypic impact of S035 is sensitive to population context.

Together, these findings evaluate their potential contribution to phenotypic variation and indicate that *AOP2* and *GSL-OH* are partially responsible for the natural variation in aliphatic GSLs in broccoli. Importantly, the contrasting outcomes across populations highlight the need for cross-population evaluation to distinguish reproducible loci from population-specific signals and underscore the relevance of these markers for understanding metabolic variation and supporting targeted improvement of GSL traits.

## 3. Discussion

GSLs play a dual role in plant defense and human health [4]. In broccoli, where florets constitute the primary edible tissue, optimizing GSL composition, particularly the relative proportions of GRA, GNA, and PRO, has become a major breeding objective. However, progress has been limited by the lack of practical and broadly applicable molecular markers for MAS. Previous genome-anchored SNP studies [3] identified loci associated with GSLs variation, but breeder-friendly markers with cross-population validation remain scarce. To address this gap, we developed KASP markers from candidate genes involved in GSL metabolism, focusing on *AOP2* and *GSL-OH*, and evaluated their performance in both a natural population and an independent F_2_ population.

Among the three core markers identified in this study, S101 showed the most consistent behavior across populations. This marker is located within the *AOP2* gene, a key enzyme in the aliphatic GSL biosynthetic pathway that catalyzes the conversion of GRA toward downstream metabolites including GNA and PRO. The significant associations observed for GNA and PRO, together with the genotype-dependent variation detected across populations, are therefore biologically consistent with the known function of *AOP2* in regulating metabolic flux within the GRA-GNA-PRO pathway. The successful validation of S101 in the independent F_2_ population further supports the robustness of this locus and suggests that variation in *AOP2* may contribute to stable modulation of aliphatic GSL composition in broccoli. From a breeding perspective, such pathway-consistent markers are particularly valuable because they provide a mechanistic basis for marker-assisted selection targeting desirable GSL profiles. Compared with prior studies that primarily focused on dissecting the genetic architecture of GSL traits within a single population [14], our work emphasizes cross-population validation and practical applicability. The significant associations of S101 with GNA and PRO, alongside genotype-dependent trends for GRA, highlight its biological relevance to the GRA-GNA-PRO pathway [11], and provide a potential molecular entry point for selective modulation of aliphatic GSL composition within defined breeding objectives. This multi-trait response pattern is consistent with metabolomic evidence indicating strong metabolic interconnections among major aliphatic GSLs.

In contrast, S074 and S035 exhibited population-dependent effects. Both markers were significantly associated with GSL traits in the natural population, but these associations were weakened or absent in the F_2_ population; S074 showed no detectable effects, and S035 retained only a trait-specific association with GNA. Such variability limits their general applicability, although these markers may still be useful for targeted improvement within a specific germplasm. These findings underscore the importance of defining the applicable scope of individual markers prior to deployment in breeding programs. The observed population-specific performance aligns with previous reports indicating that marker effectiveness in *Brassica* crops can be strongly influenced by population structure and LD patterns [14].

This study advances the development of GSL-related molecular markers for broccoli by identifying KASP markers with reproducible cross-population associations. Among the evaluated markers, S101 showed relatively stable associations across populations. It should be noted that the observed stability refers primarily to consistency across genetic backgrounds rather than across environmental conditions, which remain to be further evaluated. Therefore, its potential utility in marker-assisted selection should be considered as preliminary pending multi-environment validation. In contrast, S074 and S035 exhibited population- or trait-specific effects, indicating that their application may be more suitable for targeted breeding within defined genetic backgrounds.

Despite these promising findings, several limitations should be considered. First, the genetic diversity represented in the analyzed populations was limited. The natural population did not encompass accessions from a wide range of geographic origins, and the F_2_ population was derived from a single biparental cross, which likely resulted in low minor allele frequencies for some loci. Such constraints can reduce statistical power and limit the detection of stable marker–trait associations, as reported in previous *Brassica* studies [25]. Similar limitations have been noted in previous SNP-based GSL association studies [14]. Second, GSL accumulation is strongly influenced by environmental factors [26], including temperature, soil conditions, and developmental stage. This environmental sensitivity is well documented for GSL biosynthesis, which is regulated by complex interactions between developmental cues and abiotic factors. However, marker validation in this study was conducted under a single environmental condition. Therefore, the current conclusions should be interpreted as preliminary evidence of genetic reproducibility rather than confirmed environmental robustness. Consequently, potential gene–environment interactions affecting marker performance may not have been fully captured. Third, although the markers were developed from well-characterized candidate genes such as *AOP2*, direct functional validation was not performed, and causal relationships between specific SNPs and GSL phenotypes therefore remain to be confirmed.

Future studies should increase population size and genetic diversity by incorporating additional biparental populations and representative broccoli germplasm, which would improve minor allele frequencies and enhance marker evaluation. Validation across multiple environments and growing seasons will also be necessary to assess the stability of marker effects under variable conditions. In addition, functional validation of key loci, particularly S101, using approaches such as CRISPR/Cas9-mediated editing of *AOP2* or *GSL-OH*, would help establish causal relationships between markers and GSL traits. Integrating transcriptomic or metabolomic analyses may further clarify the molecular basis of the multi-trait associations observed in this study. Collectively, these approaches will help determine whether the identified markers can achieve robust and reproducible performance across diverse breeding contexts.

## 4. Materials and Methods

### 4.1. Plant Materials and Sample Preparation for Extracting the GSLs

The natural population used in this study consisted of 106 broccoli (*Brassica oleracea* L. var. *italica*) accessions developed and maintained by our laboratory. These materials included special germplasm resources (SG), broccoli inbred lines (BP), and hybrid cultivars (B). All plant materials are available from our laboratory upon reasonable request. For marker validation, an F_2_ population was generated from a cross between two broccoli accessions selected from the natural population that exhibited significant differences in GSLs content. All accessions of the natural population and the F_2_ population were grown under controlled greenhouse conditions at the experimental base of the Zhejiang Academy of Agricultural Sciences. When the flower heads were mature, four representative small florets were collected from both the sides and the center of each head, each approximately five centimeters in size. The florets were then placed within mesh bags and were immediately submerged in liquid nitrogen for rapid freezing. Subsequently, the frozen florets were transferred to a vacuum freeze-drier (Biosafer-18A, Biosafer Biotechnology Co., Ltd., Nanjing, China) where they were dried. Then, the dried florets were pulverized into a 40-mesh powder using a mixer mill (JXFSTPPP-24L, Shanghai Jingxin Industrial Development Co., Ltd., Shanghai, China) at 30 Hz for 1.5 min and placed into 50mL centrifuge tubes lined with desiccant at the tube bottom. The tubes were stored at −20 °C, awaiting the extraction of GSLs.

### 4.2. GSLs Extraction and Quantification

GSLs were extracted with minor modifications to our previously published protocol [23]. Briefly, approximately 200 mg of sample powder was suspended in 5 mL of boiling water and incubated for 10 min to maximize the extraction of GSLs. After centrifugation at 9000× *g* for 6 min, 1 mL of supernatant was loaded onto a 6 × 0.5 cm DEAE-Sephadex A-25 (Sigma-Aldrich, Corp., St. Louis, MO, USA), which had been activated to a height of 1 cm with 0.5 M pyridine acetate. The column was washed twice with water, once with 20 mM pyridine acetate and twice again with water. Sulphatase (1.4 U in 100 µL 0.1% solution) (Sigma-Aldrich, Corp., St. Louis, MO, USA) was introduced into the column and incubated for 16 h or overnight at room-temperature, allowing for the conversion of GSLs into their desulfo analogs. Finally, the desulfo GSLs were eluted with 1 mL of water and filtered by a 0.22 um filter(Millipore Corp., Burlington, MA, USA).

Separation was performed on a LC 2050CN HPLC (Shimadzu, Corp., Kyoto, Japan) equipped with an auto-injector and a UV-visible diode-array detector. A Shim Nex CS C18 (5 μm, 4.6 × 250 mm Shimadzu Laboratory Supplies Co., Ltd., Shanghai, China) was maintained at 30 °C and eluted with a binary gradient of water (A) and acetonitrile (B). The gradient program was: 1.5% B (5 min), linear increase to 20% B (15 min), isocratic hold (8 min), ramp to 100% B (2 min), column wash (5 min) and re-equilibration to 1.5% B (3 min). The flow rate was 1.0 mL/min. Ortho-nitrophenyl-β-D-galactopyranoside (ONPG, Sigma-Aldrich, Corp., St. Louis, MO, USA) was used as the internal standard for the calibration of GSL retention times. No commercial pure GSL standards were used in this study. Individual GSLs were identified by comparing their retention times and relative elution order with those of *Arabidopsis thaliana* ecotype Col-0 leaf GSLs [27] and further confirmed by their characteristic retention behavior under the established chromatographic conditions. GSL concentrations were calculated from HPLC peak areas at 226 nm using published UV response factors for individual desulfo GSLs, and the results are expressed as μ mol/g dry weight [28]. The limit of detection (LOD) for each individual GSL compound was determined based on a signal-to-noise ratio of 3 (S/N = 3). Concentrations below the LOD were recorded as 0.00 and included in subsequent statistical analyses.

### 4.3. Development of KASP Markers for GSL-Related Genes in Broccoli

#### 4.3.1. Primer Design

Whole-genome re-sequencing data of 23 core broccoli varieties (20× coverage) were obtained from our previous study [16]. The sequencing reads were aligned to the HDEM reference genome [20]. After quality control, SNP loci were identified. Based on the genomic positions of SNPs and the functional annotation of their host genes, SNPs potentially associated with the GSLs metabolic pathway in broccoli were predicted. To minimize marker development costs, a representative marker was selected from redundant markers that show consistent genotyping results across the 23 core broccoli varieties. If this representative marker failed to produce high-quality genotyping, an alternative redundant marker was selected for a second round of primer design until successful application.

For each targeted locus, 50 bp flanking sequences upstream and downstream were extracted using TB-tools (Version 1.0; https://github.com/CJ-Chen/TBtools, accessed on 10 January 2026) software [29]. These sequences were submitted to the LGC Primer Design website (https://www.biosearchtech.com, accessed on 10 January 2026) for KASP assay design. All primers were designed to contain at least 30% GC content. All designed primers were validated using the Integrative Genomics Viewer (IGV, Version 2.17.2; https://software.broadinstitute.org/software/igv/, accessed on 10 January 2026) to confirm the absence of additional variants within the 50 bp flanking regions. Alleles were flipped to the forward strand using complementary base pairing where necessary. As a result, we obtained 108 KASP markers covering nearly all GSLs metabolism-related genes in broccoli. Ultimately, 97 high-quality genotyped KASP markers were employed for phenotypic association analysis. Each primer set consists of two allele-specific forward primers (FAM and HEX labeled) and one common reverse primer. The 3′ ends of the two forward primers contain the two allelic-specific SNP bases, while the 5′ ends contain the fluorescent sequence tags FAM (5′-GAAGGTGACCAAGTTCATGCT-3′) and HEX (5′-GAAGGTCGGAGTCAACGGATT-3′). Detailed primer information is provided in Table S15.

#### 4.3.2. KASP Marker Genotyping

Total genomic DNA was extracted from fresh leaf tissues using an optimized cetyltrimethylammonium bromide (CTAB) protocol [30]. The quality and concentration of the DNA were assessed using a Nano Drop 2000 spectrophotometer (Thermo Fisher Scientific, Waltham, MA, USA). KASP genotyping assays were performed at the Shared Instrumentation Platform of the Zhejiang Academy of Agricultural Sciences Public Laboratory based on previous methods and making adjustments [16], using the LGC high-throughput genotyping system (LGC Biosearch Technologies, Hoddesdon, UK) and genotyping of 96-well plates. The final reaction volume is 10.14 µL. KASP Reaction Mixture contained about 5.0 µL KASP PCR master mix, 0.14 µL Primer mix (5 nM) and 5.0 µL Template DNA (20 ng/µL). The PCR reaction procedure was consistent with that used in previous studies [16]. Following PCR amplification, fluorescence signals were detected using a FRET-capable plate reader (Molecular Devices, Sunnyvale, CA, USA). Fluorescence data were analyzed using Kluster Caller TM software (Version 3.4.1.36) (LGC Biosearch Technologies, Hoddesdon, UK) to visualize allelic discrimination and assign genotypes.

### 4.4. Association Analysis Between KASP Markers and GSL Traits

Phenotypic data for 13 GSL traits were collected in the natural population (Table S3) and association analyses were conducted for all traits. For clarity and focused interpretation, six representative GSL components are presented in detail in the main text, while complete results for all traits are provided in the Supplementary Materials. These traits were prioritized based on their key positions in the GSL biosynthetic pathway, clear phenotypic distributions, and overall interpretability of association patterns across analytical models. In the F_2_ population, 11 GSL traits were measured. Four representative traits are shown in the main text for direct comparison with the natural population, while the others were either not detected in the natural population or showed weak signals which are included in the Supplementary Materials.

Association analysis was performed using both a GLM and a MLM implemented in TASSEL (Version 5.0; Bioinformatics Research Center, Iowa State University, Ames, IA, USA). [24]. The GLM was initially applied for marker–trait association screening. SNPs were considered candidate loci when they satisfied a nominal significance threshold of *p* < 0.05 and explained more than 5% of the phenotypic variance (*R*^2^ > 5%). To account for population structure and relatedness, the MLM incorporated the first three principal components (PC1–PC3) and a kinship matrix (K) calculated using the centered identity-by-state (IBS) method (Q + K model).

To account for potential multiple testing effects, FDR correction was applied using the BH procedure, and adjusted *q*-values were calculated. Association results were interpreted by jointly considering nominal *p*-values, FDR-adjusted significance levels, and consistency across analytical models. Given the candidate gene-based design and the relatively small number of tested SNP markers, marker prioritization was based on integrated evidence rather than relying solely on a strict FDR-adjusted threshold. Manhattan [31] and QQ plots [32] were generated to visualize association signals and assess potential inflation. For graphical reference, nominal (*p* = 0.05) and stringent (*p* = 1 × 10^−5^) thresholds were plotted in Manhattan plots. SNP density plots [33] were generated to assess the chromosomal distribution of developed KASP markers. LD analysis was performed using PLINK (Version 1.9; Chang CC, et al., Cambridge, MA, USA) [34], with pairwise R^2^ and D’ values calculated to evaluate marker redundancy and chromosomal distribution.

Given the non-normal distribution in GSL traits, phenotypic normality was assessed using the Shapiro–Wilk test [35], and non-parametric statistical methods were applied. Differences between two homozygous genotype classes were analyzed using the Mann–Whitney U test [36], while comparisons among three genotypic classes were conducted using the Kruskal–Wallis H test [37]. Statistical significance was defined at *p* < 0.05. For genotype effect interpretation, cutoffs determined by ROC curve analysis were used to distinguish high- and low-content genotypes. Correlations among GSL traits were assessed using Spearman’s rank correlation coefficients [38]. All genotype-based statistical analyses and data visualization were performed using GraphPad Prism (Version 10.5.0; GraphPad Software, San Diego, CA, USA [39] and R (Version 4.5.1; R Foundation for Statistical Computing, Vienna, Austria) [40].

## 5. Conclusions

This study developed GSL-related KASP markers through candidate gene-based SNP screening combined with cross-population validation. Among the evaluated markers, S101 showed consistent associations with GNA and PRO in both the natural population and the independent F_2_ population, accompanied by clear homozygous genotype-dependent phenotypic differentiation. These results suggest that S101 is a promising candidate marker for further validation in marker-assisted selection targeting GSL composition. In contrast, S074 and S035 exhibited population- or trait-dependent associations, indicating that their effects are influenced by genetic background and allele distribution. This variability highlights that statistical significance observed in a single population is insufficient to ensure breeding applicability and underscores the importance of validation in genetically distinct populations. Overall, this study underscores the importance of multi-population validation for KASP markers for complex metabolic traits such as GSL content and provides a foundation for future assessment of the effect magnitude, environmental stability, and practical utility prior to large-scale implementation in broccoli and related *Brassica* breeding programs.

## Figures and Tables

**Table 1:** The characteristics of the contents of GSLs in the florets of broccoli in nature population (μ mol/g).

GSLs	Mean ± SD	Range	CV
PRO	1.57 ± 2.98	0.00–13.85	1.89
GRA	5.63 ± 3.87	0.18–25.20	0.69
SIN	0.28 ± 1.05	0.00–6.56	3.78
GNA	0.24 ± 0.49	0.00–2.69	2.08
4HGBS	1.09 ± 0.78	0.13–4.38	0.72
4MGBS	2.61 ± 1.19	0.90–8.61	0.46

GSLs abbreviations, PRO: progoitrin; GRA: glucoraphanin; SIN: sinigrin; GNA: gluconapin; 4HGBS: 4-hydroxyglucobrassicin; 4MGBS: 4-meth-oxyglucobrassicin; SD: standard deviation, CV: coefficient of variation. Values equal to 0.00 indicate concentrations below the limit of detection (LOD) and were included in statistical analyses. Data are presented for the natural population (*n* = 106).

**Table 2:** The characteristics of the contents of GSLs in the florets of broccoli in F_2_ population (μ mol/g).

GSLs	Mean ± SD	Range	CV
PRO	1.78 ± 1.92	0.00–8.65	1.08
GRA	5.86 ± 2.62	0.96–14.81	0.45
GNA	0.82 ± 1.51	0.00–8.29	1.85
4HGBS	1.17 ± 0.43	0.04–3.00	0.37

GSLs abbreviations, PRO: progoitrin; GRA: glucoraphanin; GNA: gluconapin; 4HGBS: 4-hydroxyglucobrassicin; SD: standard deviation; CV: coefficient of variation. Values equal to 0.00 indicate concentrations below the LOD and were included in the statistical analyses. Data are presented for the F_2_ population (*n* = 189).

**Table 3:** The accuracy of S101 and S035 KASP markers prediction in F_2_ population.

KASP Marker	Candidate Gene	Chr.	Pos	Phenotype	H_G	L_G	H_A	L_A
S101	*AOP2*		1,616,635	PRO	AA	GG	89.80%	97.67%
		C9		GNA	AA	GG	93.88%	90.70%
				GRA	GG	AA	87.55%	58.14%
				4HGBS	GG	AA	65.12%	65.31%
S035	*GSL-OH*	C3	21,810,653	GNA	CC	TT	60.53%	65.63%

H_G: high-content genotype, L_G: low-content genotype, H_A: high-content prediction accuracy, L_A: low-content prediction accuracy. Data are presented for the F_2_ population (*n* = 189). For marker S101, the sample sizes were 49 for the AA genotype and 43 for the GG genotype. For marker S035, the sample sizes were 64 for the TT genotype and 38 for the CC genotype. Pos, physical position on the chromosome.

## Data Availability

The 23 re-sequencing samples in this study are available from the National Center for Biotechnology Information (NCBI) at https://www.ncbi.nlm.nih.gov/ (accessed on 10 January 2026) under the accession number PRJNA681704.

## Supplementary Materials

The following supporting information can be downloaded at: https://www.mdpi.com/article/10.3390/ijms27062714/s1.

## Abbreviations

The following abbreviations are used in this manuscript:

BH	Benjamini–Hochberg
CTAB	Cetyltrimethylammonium bromide
CV	Coefficient of variation
FDR	False discovery rate
GSLs	Glucosinolates
GRA	Glucoraphanin
GNA	Gluconapin
GLM	General linear model
HPLC	High-performance liquid chromatography
IBS	Identity-by-state
IQR	Interquartile range
IGV	Integrative Genomics Viewer
KASP	Kompetitive Allele-Specific PCR
LOD	Limit of detection
LD	Linkage disequilibrium
MAS	Marker-assisted selection
MLM	Mixed linear model
ONPG	Ortho-nitrophenyl-β-D-galactopyranoside
PRO	Progoitrin
PVE	Phenotypic variance explained
QQ	Quantile-quantile
ROC	Receiver operating characteristic
SNPs	Single-nucleotide polymorphisms
SIN	Sinigrin
SD	Standard deviation
4HGBS	4-hydroxyglucobrassicin
4MGBS	4-methoxyglucobrassicin
