# Supplementary material for: Development and Application of KASP Markers for Candidate Glucosinolate Biosynthesis Genes in Broccoli
Source: Int J Mol Sci. 2026 Mar 16;27(6):2714. doi: 10.3390/ijms27062714 (PMC13026235; doi:10.3390/ijms27062714)
Supplement: Supplementary file 1 [file ijms-27-02714-s001.zip › supplement figure.pdf]

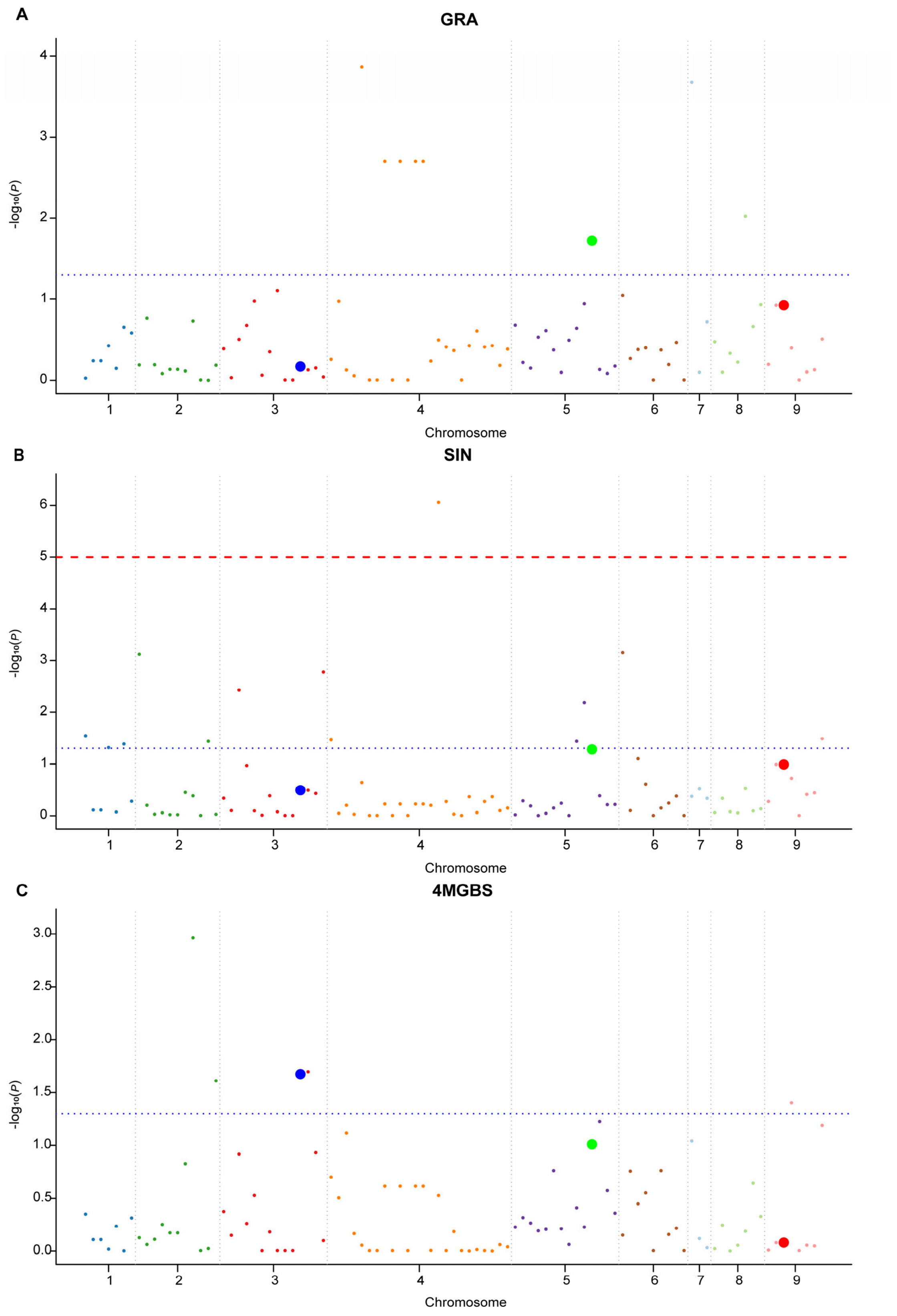

Figure S1. Manhattan plots of candidate gene-based association analysis for GRA, SIN, and 4MGBS contents in 106 broccoli accessions in GLM. (**A**) GRA; (**B**) SIN; (**C**) 4MGBS. The x-axis represents the physical positions of SNPs along the nine broccoli chromosomes (bp), and the y-axis represents the -log10(*P*) values. Each point represents a SNP, and there are a total of 97 SNPs. Alternating colors are used

to distinguish the nine broccoli chromosomes for better visualization. Red bold dots represent S101, green bold dots represent S074, and blue bold dots represent S035. Two significance thresholds are shown: a blue dashed line indicates $P = 5 \times 10^{-2}$, and a red dashed line indicates $P = 1 \times 10^{-5}$. Detailed association results including chromosomal position, P values, and proportion of phenotypic variance explained are provided in Table S5.

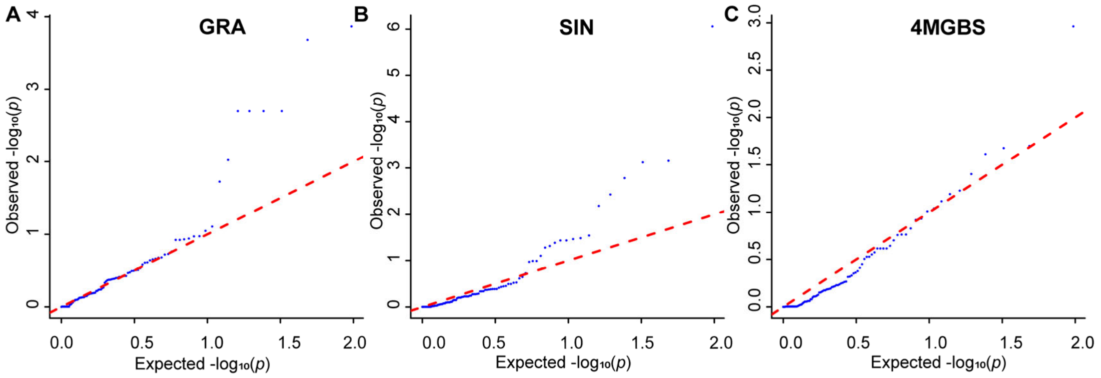

Figure S2 QQ plots of 97 SNPs associated with GRA, SIN, and 4MGBS. (**A**) GRA; (**B**) SIN; (**C**) 4MGBS. Each point represents an SNP, and there are a total of 97 SNPs. The x-axis represents the expected -$\log_{10}(p)$ values, and the y-axis represents the observed -$\log_{10}(p)$ values. The blue dots represent the observed association signals, and the red line represents the theoretical null distribution (y = x). Comparing observed and expected -$\log_{10}(p)$ values to assess the overall distribution of association signals. Detailed association statistics are provided in Table S5.

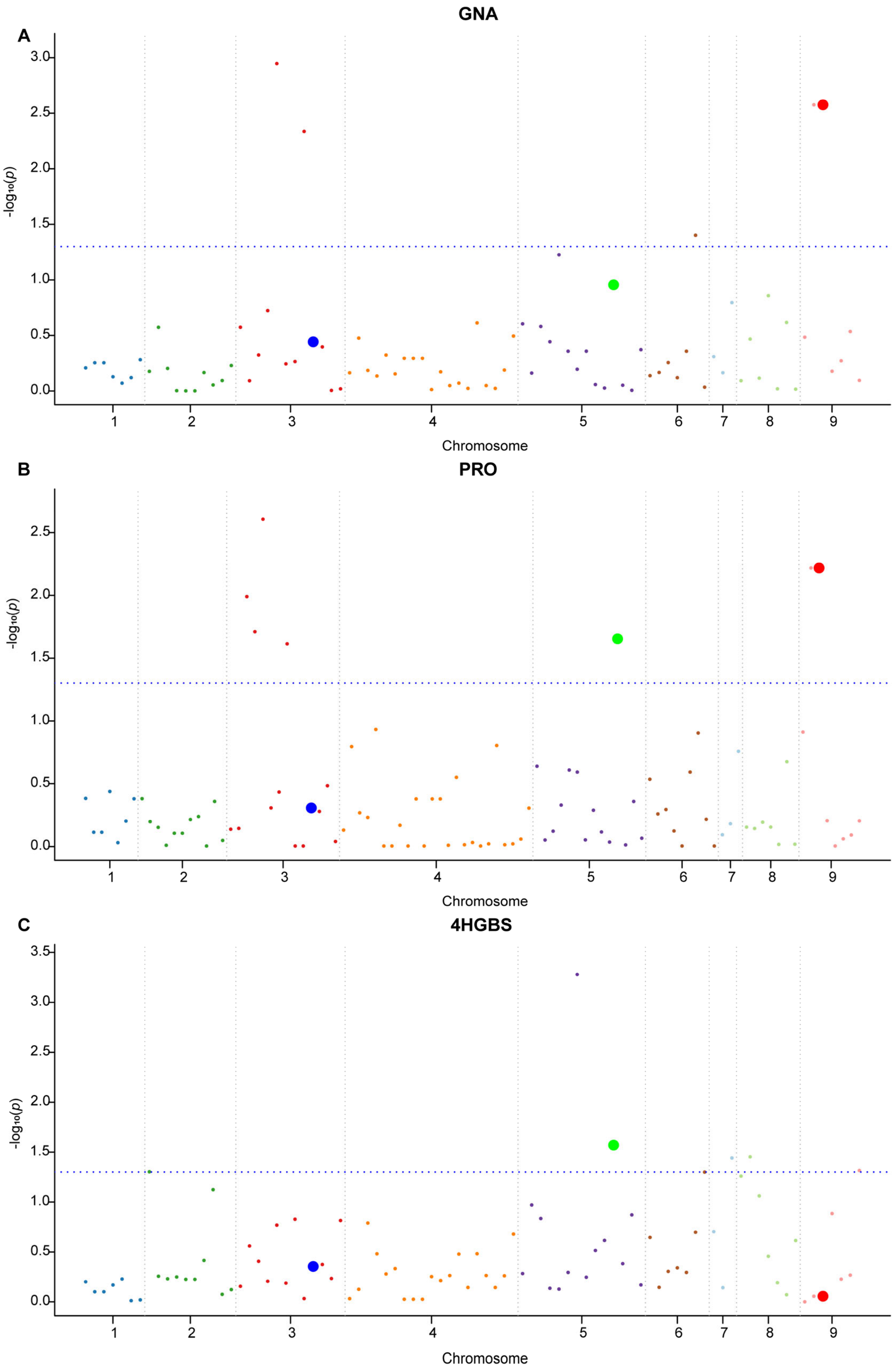

Figure S3. Manhattan plots of candidate gene-based association analysis for GNA, PRO, and 4HGBS contents in 106 broccoli accessions in MLM. **(A)** GNA; **(B)** PRO; **(C)** 4HGBS. The x-axis represents the physical positions of SNPs along the nine broccoli chromosomes (bp), and the y-axis represents the -

$log_{10}(p)$ values. Each point represents an SNP, and there are a total of 97 SNPs. Alternating colors are used to distinguish the nine broccoli chromosomes for better visualization. Red bold dots represent S101, green bold dots represent S074, and blue bold dots represent S035. Two significance thresholds are shown: a blue dashed line indicates $P = 5 \times 10^{-2}$, and a red dashed line indicates $P = 1 \times 10^{-5}$. Detailed association results including chromosomal position, P values, and proportion of phenotypic variance explained are provided in Table S7.

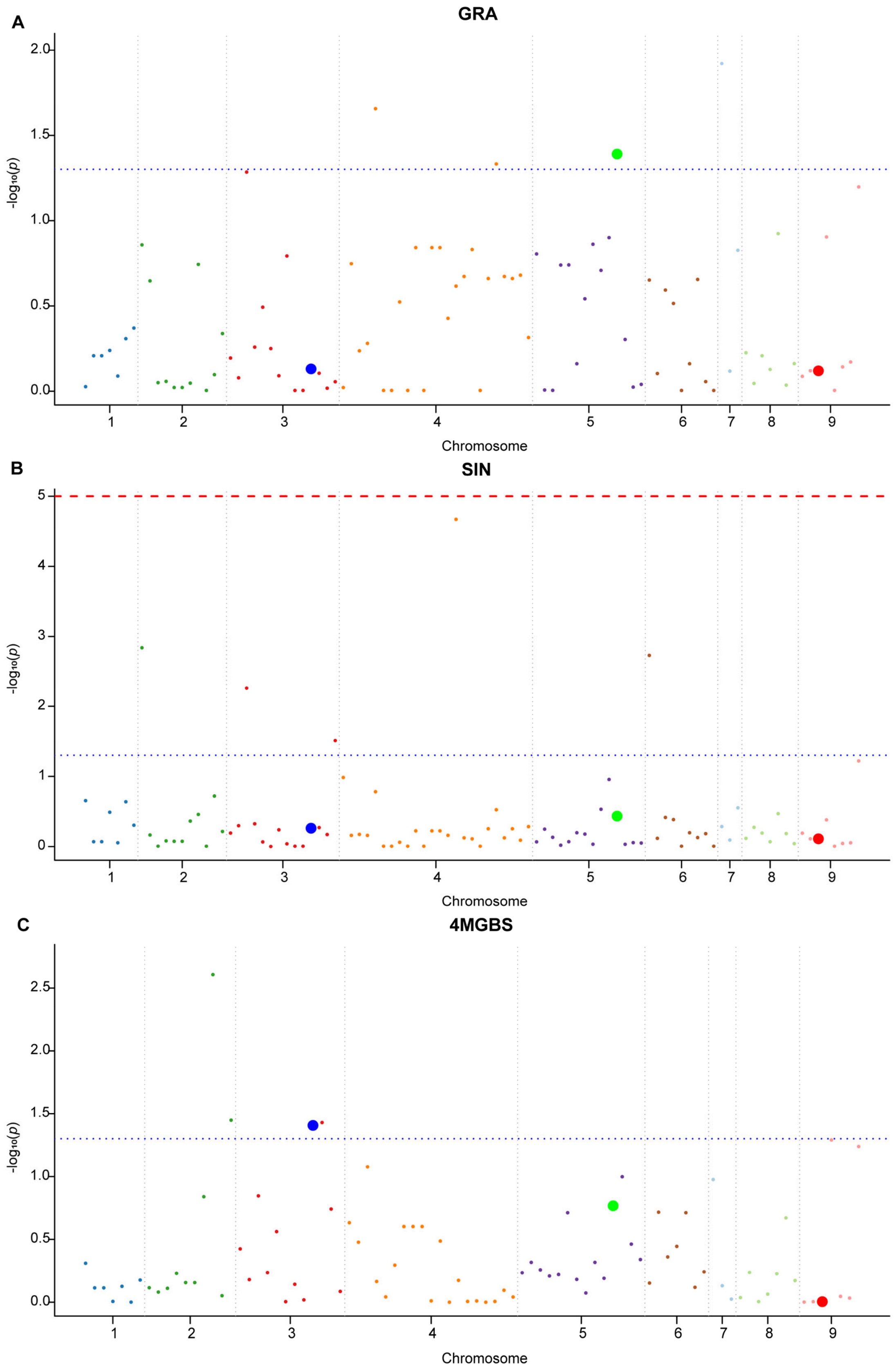

Figure S4. Manhattan plots of candidate gene-based association analysis for GRA, SIN, and 4MGBS contents in 106 broccoli accessions in MLM. (**A**) GRA; (**B**) SIN; (**C**) 4MGBS. The x-axis represents the physical positions of SNPs along the nine broccoli chromosomes (bp), and the y-axis represents the -

log10(*p*) values. Each point represents an SNP, and there are a total of 97 SNPs. Alternating colors are used to distinguish the nine broccoli chromosomes for better visualization. Red bold dots represent S101, green bold dots represent S074, and blue bold dots represent S035. Two significance thresholds are shown: a blue dashed line indicates $P = 5 \times 10^{-2}$, and a red dashed line indicates $P = 1 \times 10^{-5}$. Detailed association results including chromosomal position, *P* values, and proportion of phenotypic variance explained are provided in Table S7.

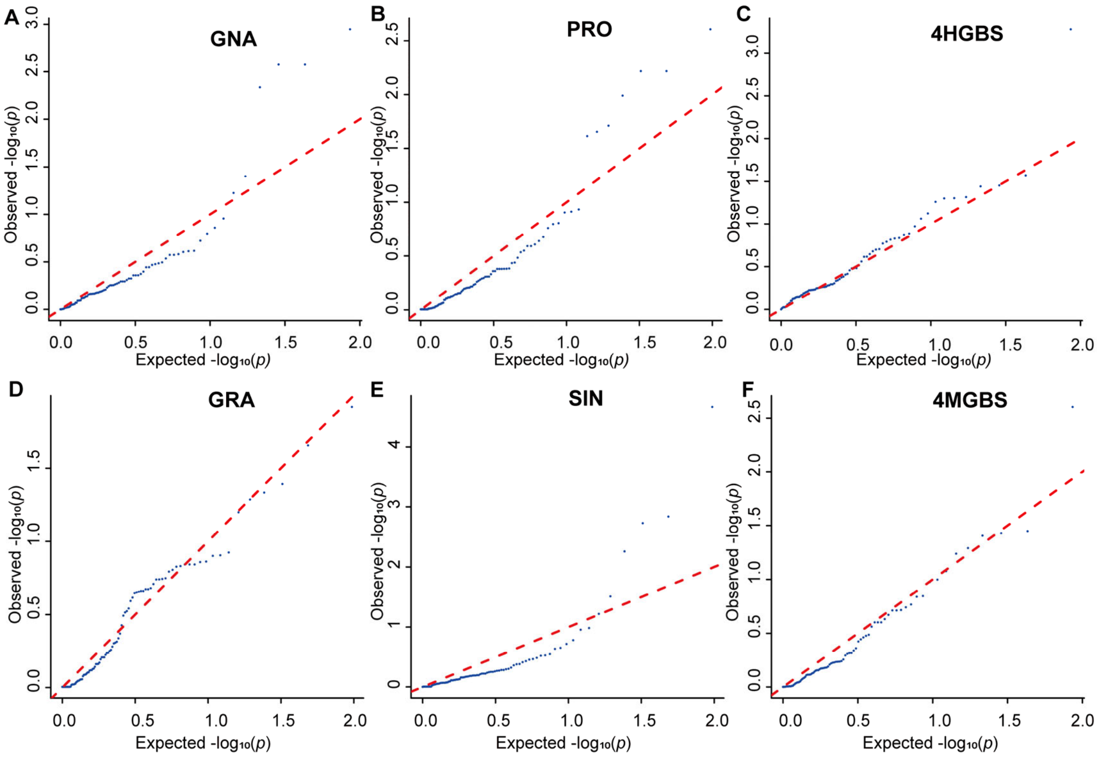

Figure S5 QQ plots of 97 SNPs associated with GNA, PRO, 4HGBS, GRA, SIN, and 4MGBS in MLM. (**A**) GNA; (**B**) PRO; (**C**) 4HGBS; (**D**) GRA; (**E**) SIN; (**F**) 4MGBS. Each point represents an SNP, and there are a total of 97 SNPs. The x-axis represents the Expected $-\log_{10}(p)$ values, and the y-axis represents the Observed $-\log_{10}(p)$ values. The blue dots represent the observed association signals, and the red line represents the theoretical null distribution (y = x). Comparing observed and expected $-\log_{10}(p)$ values to assess the overall distribution of association signals. Detailed association statistics are provided in Supplementary Table S7.

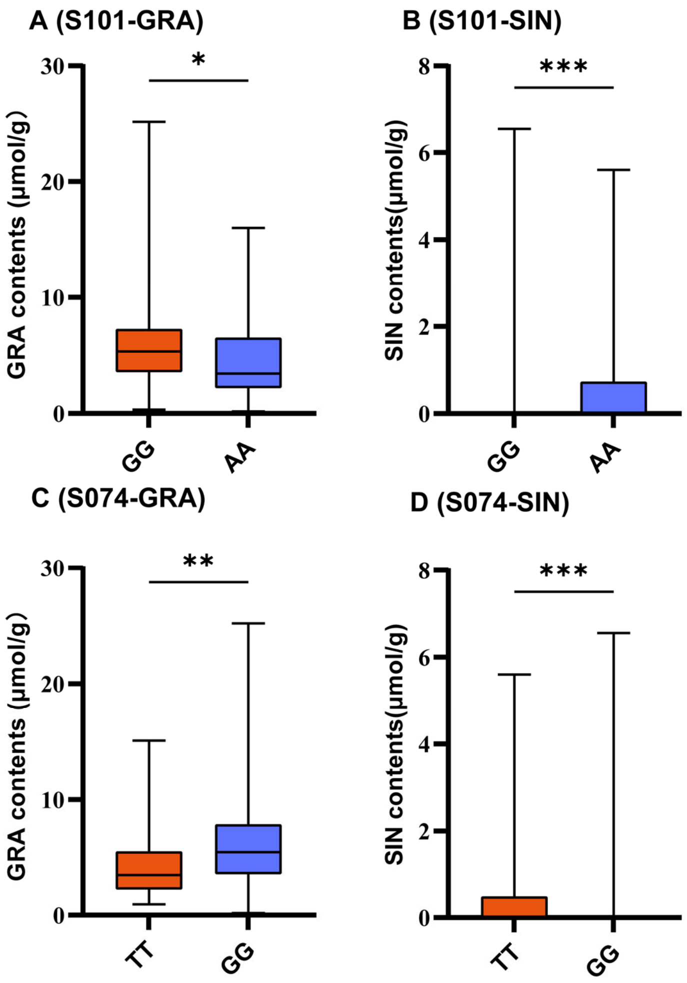

Figure S6. Genotype-phenotype associations of key KASP markers in a natural broccoli population. (**A-B**) Associations of S101 with GRA and SIN content. (**C-D**) Associations of S074 with GRA and SIN content. S101: Chr9: 1,616,635; S074: Chr5: 2,407,717. Data are presented for the natural population (*n* = 106). Boxes represent the inter quartile range (IQR), horizontal lines indicate median values, and whiskers denote the minimum and maximum values. Different letters indicate significant differences among genotypes. Significance levels are indicated as: *, P ≤ 0.05; **, P ≤ 0.01; ***, P ≤ 0.001. Detailed statistics are provided in Table S11.

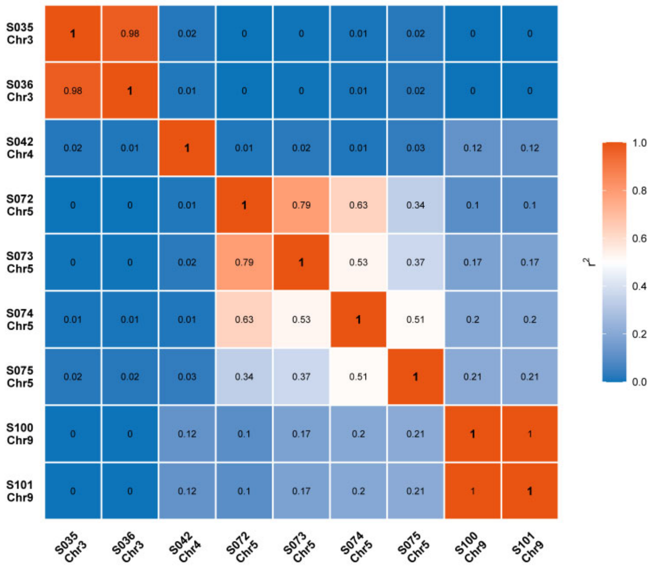

Figure S7 Linkage disequilibrium heatmap showing pairwise r^2 values among nine candidate SNPs. Both axes represent SNP identifiers and their chromosomal positions. The color gradient ranges from blue ($r^2 = 0$) to red ($r^2 = 1$), indicating increasing LD strength. Exact r^2 values are displayed within each cell. Pairwise LD coefficients (r^2 and D′) were calculated using PLINK v1.9; corresponding D′ values are provided in Table S10..

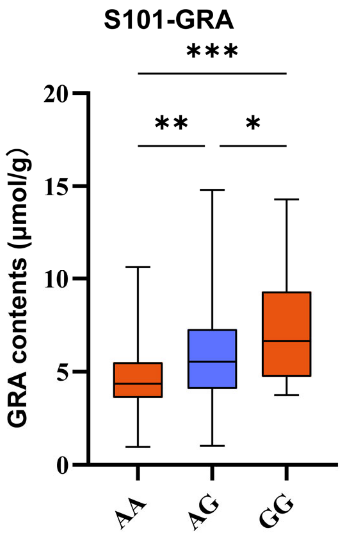

Figure S8 Significant associations between S101 genotypes and GRA in a F_2 broccoli population. S101: Chr9: 1,616,635. Data are presented for the F_2 population ($n = 189$). Boxes represent the inter quartile range (IQR), horizontal lines indicate median values, and whiskers denote the minimum and maximum values. Different letters indicate significant differences among genotypes. Significance levels are indicated as: *, $P \leq 0.05$; **, $P \leq 0.01$; ***, $P \leq 0.001$. Detailed statistics are provided in Table S12.

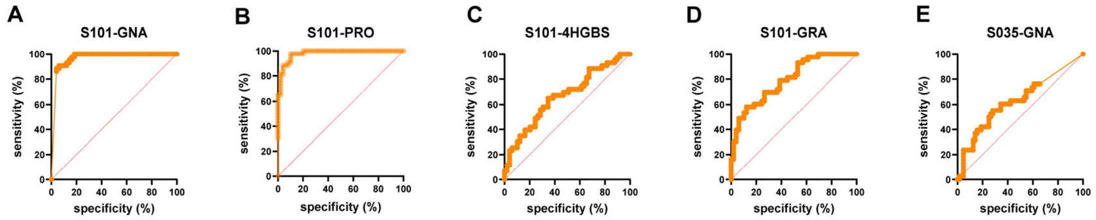

Figure S9 Receiver Operating Characteristic Curve analysis of various indicators. (**A-D**) the ROC curves indicators of S101. (A) S101-GNA, (B) *S*101-PRO, (C) *S*101-4HGBS, (D) S101-GRA; (E) the ROC curves indicators of S035-GNA. The x-axis represents specificity (%), and the y-axis represents sensitivity (%). ROC curves were used to evaluate the predictive efficacy of each indicator for the target phenotype; the closer the curve is to the upper left corner, the higher the predictive value.
